# Crumbs proteins control ciliogenesis and centrosome organization: what about the mechanism?

**DOI:** 10.1186/2046-2530-4-S1-P23

**Published:** 2015-07-13

**Authors:** M Barthelemy-Requin, JP Chauvin, CL Baron-Gaillard, P Rashbass, D Massey-Harroche, A Le Bivic

**Affiliations:** 1 IBDM, Université Aix-Marseille, CNRS UMR 7288, Campus de Luminy, Case 907, Marseille, France; 2 Department of Biomedical Sciences, University of Sheffield, Sheffield, UK

## Objective

This project aims to identify the function of polarity protein complexes in the formation of the primary cilia. We focus specifically on the two apical complexes: the Crumbs (Crb/Pals-1/PATJ) and the PAR (Par3/Par6/aPKC) complexes. In this study, we investigated the role of Crumbs in ciliogenesis.

## Methods

The depletion of crumbs2 (Crb2) and/or crumbs3 (Crb3) via transient siRNA transfection in ARPE-19 cells (human pigmented retina epithelium) leads to strong inhibition of ciliogenesis which underscores the involvement of Crb2 and Crb3 in the formation of the primary cilia.

## Results

Crb2 and Crb3 share many protein interactions and mechanisms of compensation could exist. However our data revealed the contrary since we showed that primary cilia formation requires a threshold level of both Crb2 and Crb3. To decipher the mechanism that underlies this requirement we have focused on Crb2, which is predominantly expressed in ARPE-19 cells. Using both optical imaging and electron microscopy we showed that Crb2 is involved in cilia initiation but not cilia maintenance. Furthermore we uncovered that Crb2 acts at a very early stage of ciliogenesis, by affecting the localization of centriolar and peri-centriolar markers such as PCM-1.

## Conclusion

Crb2 allows the efficient organization of the centrosome and associated proteins and the primary vesicle formation to promote ciliogenesis. Taken together, our data show that Crb2 is essential for the primary ciliogenesis by a still unknown mechanism.

